# A Combinational Clustering Based Method for cDNA Microarray Image Segmentation

**DOI:** 10.1371/journal.pone.0133025

**Published:** 2015-08-04

**Authors:** Guifang Shao, Tiejun Li, Wangda Zuo, Shunxiang Wu, Tundong Liu

**Affiliations:** 1 Department of Automation, Xiamen University, Xiamen, P.R. China; 2 Information Engineering College, Jimei University, Xiamen, P.R. China; 3 Department of Civil, Architectural and Environmental Engineering, University of Miami, Coral Gables, United States of America; Queen's University Belfast, UNITED KINGDOM

## Abstract

Microarray technology plays an important role in drawing useful biological conclusions by analyzing thousands of gene expressions simultaneously. Especially, image analysis is a key step in microarray analysis and its accuracy strongly depends on segmentation. The pioneering works of clustering based segmentation have shown that k-means clustering algorithm and moving k-means clustering algorithm are two commonly used methods in microarray image processing. However, they usually face unsatisfactory results because the real microarray image contains noise, artifacts and spots that vary in size, shape and contrast. To improve the segmentation accuracy, in this article we present a combination clustering based segmentation approach that may be more reliable and able to segment spots automatically. First, this new method starts with a very simple but effective contrast enhancement operation to improve the image quality. Then, an automatic gridding based on the maximum between-class variance is applied to separate the spots into independent areas. Next, among each spot region, the moving k-means clustering is first conducted to separate the spot from background and then the k-means clustering algorithms are combined for those spots failing to obtain the entire boundary. Finally, a refinement step is used to replace the false segmentation and the inseparable ones of missing spots. In addition, quantitative comparisons between the improved method and the other four segmentation algorithms--edge detection, thresholding, k-means clustering and moving k-means clustering--are carried out on cDNA microarray images from six different data sets. Experiments on six different data sets, 1) Stanford Microarray Database (SMD), 2) Gene Expression Omnibus (GEO), 3) Baylor College of Medicine (BCM), 4) Swiss Institute of Bioinformatics (SIB), 5) Joe DeRisi’s individual tiff files (DeRisi), and 6) University of California, San Francisco (UCSF), indicate that the improved approach is more robust and sensitive to weak spots. More importantly, it can obtain higher segmentation accuracy in the presence of noise, artifacts and weakly expressed spots compared with the other four methods.

## Introduction

DNA Microarray is a powerful tool for biologists to simultaneously analyze thousands of genes [1]. Microarray technology becomes useful in many fields such as the diagnosis of disease, biomedicine, gene discovery, drug discovery and so on [2]. DNA microarray analysis aims to identify different gene expressions which can be used in studying the function of genes [3]. Generally, microarray technology includes experimental design, RNA and probe preparation, hybridization to DNA arrays, image processing, data analysis, biological confirmation and microarray databases [4–6]. First of all, Ribonucleic Acids (RNAs) are isolated from the experimental sample and control sample. Secondly, these extracted RNAs are converted into cDNAs. Subsequently, the mixture of these cDNAs, labeled with fluorescent dyes Cy3 (Green) and Cy5 (Red), is then hybridized to a glass slide. Finally, the slide is scanned with red and green laser and two different channels of array images are obtained [7]. Actually, the difference in fluorescence between these two color channels shows the relative difference of the gene’s expression between those two sources (experimental and control). Therefore, the microarray image processing plays an important role in extracting a series of meaningful biological conclusions regarding gene expression.

Generally speaking, microarray image processing is comprised of three major steps: 1) gridding, 2) segmentation and 3) intensity extraction. Gridding aims to separate each spot into a single area by segmenting the image into numerous compartments. Segmentation usually classifies the pixels in a region immediately surrounding the gene as belonging to either the foreground or background domains [3]. Intensity extraction tends to calculate the red and green foreground intensity pairs and background intensities [8]. Thus, a higher precision of each image processing step is needed for extracting accurate gene expression values and meaningful biological application. Especially, segmentation, as a previous step to intensity extraction, imposes a significant effect on the accuracy of image processing.

**Fig 1:**
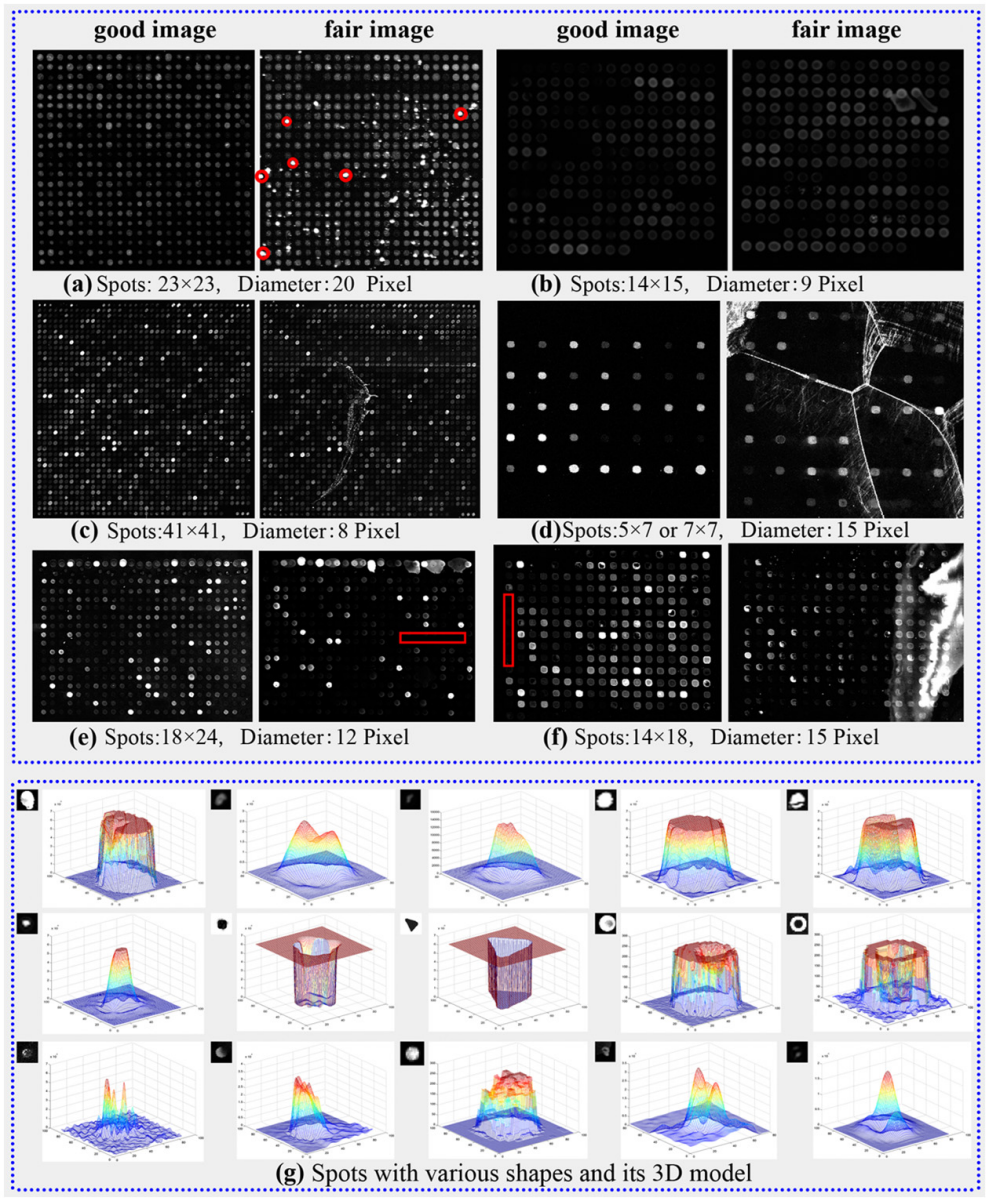
Sub-grids with various quality on data set of (a) BCM, (b) UCSF, (c) DeRisi, (d) SIB, (e) GEO, (f) SMD, and (g) spots with various shapes.

However, the complex preparation procedure of microarray, including the manufacture of cDNA microarray chip, hybridization of mRNA extracted from the sample, and scanning of chips may introduce serious errors. These facts demand the compensation of defects to the image processing procedures. To the best of our knowledge, an ideal microarray image would be characterized by deterministic grid geometry, known background intensity with zero uncertainty, pre-defined spot shape, and constant spot intensity [9]. Yet the actual microarray images may contain thousands of spots of various sizes, shapes, and intensity levels. They also contain inhomogeneous background and are contaminated by noise and artifacts. Fig 1 exhibits the typical difficulties in microarray image processing.

According to our knowledge, the diverse image content comes from the following three aspects:

1. As for the slides, the numbers of grid lines and spots are various towing to the different chip manufacturers. The image and spot resolutions are also different, such as one image in BCM data set (see Fig 1a) with the image resolution of 4,325×11,612 and spot of 25×25(pixel). However, the resolutions of image and spot are 1,910×5,550 and 18×18 respectively for one image in SMD data set (see Fig 1f). Moreover, the image quality sometimes is high or poor. Here the poor image quality is defined as that image with lower contrast and may contain noise and missing spots.
2. In terms of sub-grid, there is a non-uniform distribution, e.g., some sub-grids in DeRisi data set (see Fig 1c) are compact while others in SIB data set may be sparse (see Fig 1d). In addition, the sub-grid may contain noises with various types and levels. The missing spots also exist to varying degrees (see Fig 1e).
3. According to spot, its shapes vary from circle to square to triangle, depending on the gene chip companies. If the basic shape is circle, there may exist the “peak-shaped spot, doughnut-shaped spot, egg-shaped spot or volcano-shaped spot” as Fig 1g shown [10]. Furthermore, some spots may be stuck together.

Microarray image reveals that various qualities, as mentioned above, can be attributed to 1) different physical and chemical conditions during construction stage, 2) the laser light reflection, 3) photon noise and electronic noise introduced during scanning, and 4) dust on the glass slide [11]. These impairments will affect the cDNA microarray image formation and make the image processing be indeed complicated and challenging. Moreover, segmentation of spots can be further complicated in the non-uniform shape and surface intensity distribution. To eliminate the processing errors from propagating further down the processing pipeline to the gene expression analysis tasks, more accurate and sophisticated segmentation methods are needed [12].

Until now, except for those pioneering works with thresholding [13] and shape-based [14] methods, new algorithms are constantly emerging, such as mathematical morphological [15], genetic algorithm [16], artificial neural network [17], Markov random field [18], wavelet [19], clustering [20], and support vector machines [21].

Actually, each method has its advantages and disadvantages, and there is no one algorithm that can tackle all the microarray image segmentation problems perfectly. What’s more, the most recent and state-of-the-art work for automatic image segmentation is clustering based algorithms. Compared to the hierarchical clustering, the partition clustering is simple and it includes several algorithms, such as k-means, k-medoids, k-modes, k-prototypes, fuzzy c-means and so on. Among those the k-means clustering algorithm, which classifies the objects into k number of group based on features, is the most simple and fast method even though it is sensitive to the noises [2]. However, the k-medoids approach is another classical partitioning method by selecting one point in cluster as representative instead of the cluster center in k-means [22]. But it suffers from the serious drawback that its performance heavily depends on the initial starting conditions. K-modes approach is an extending of k-means by replacing the means of clusters with modes. Furthermore, k-prototypes method integrates the k-means and k-modes algorithm to cluster the mixed data by using k-modes approach to update the categorical attribute values of cluster prototypes [23]. In addition, the fuzzy c-means clustering method is also introduced to dealing with some uncertainty problems, i.e. they considered that each pixel may belong to more than one cluster [24]. However, fuzzy c-means clustering algorithm strongly depends on the fuzziness parameter and also sensitive to noises. Recently, to minimize the affect of noise, a moving k-means clustering algorithm is proposed based on k-means clustering by introduced fitness function [25]. Considering that most of the partitional clustering approaches are based on k-means, and moving k-means clustering can minimize the noise affect, this paper introduces an idea of integrating both k-means clustering and moving k-means clustering to conduct the segmentation.

Therefore, to improve the segmentation accuracy, in this article we propose an adaptive segmentation method by combining the k-means and moving k-means clustering methods. This method is unique in the way to enhance the image contrast automatically and well-performed on different data sets.

The rest of the paper is organized as follows. Section II makes an insight into the related works. A framework of the proposed method which combines k-means clustering method and moving k-means clustering method is discussed in Section III. Contrast enhancement is also introduced. Selected cDNA Microarray images from six data sets are used for experiments. Comparisons with the prior art in cDNA image segmentation are also provided in Section IV. Finally, the main conclusions are summarized in Section V.

## Related Works

**Fig 2:**
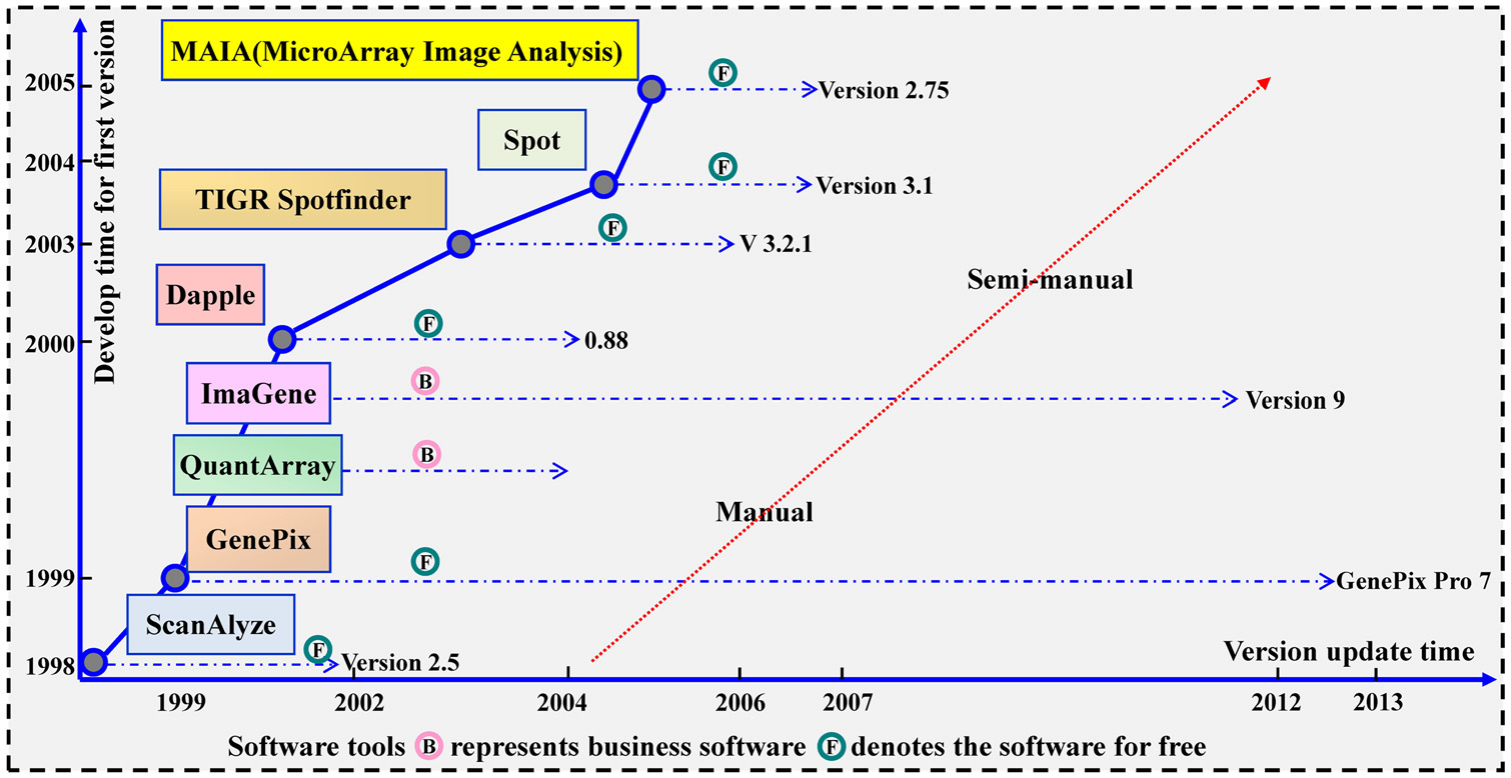
Software tools for microarray image processing.

In the last few years, several commercial softwares and freeware packages have been built for microarray image processing. As shown in Fig 2, most of these tools are manual or semi-manual and a series of image processing technologies have been proposed. Fixed circle segmentation has been used in ScanAlyze (Eisen 1999), adaptive circle segmentation has been applied in GenePix (Axon Instruments, Inc. 1999), seeded region growing (SRG) algorithm has been employed in Spot (Buckly 2000), threshold based method has been used in QuantArray (GSI Lumonics 1999), gradient based spot segmentation employed in Dapple, and a histogram-based segmentation method has been applied in ImaGene [26].

**Fig 3:**
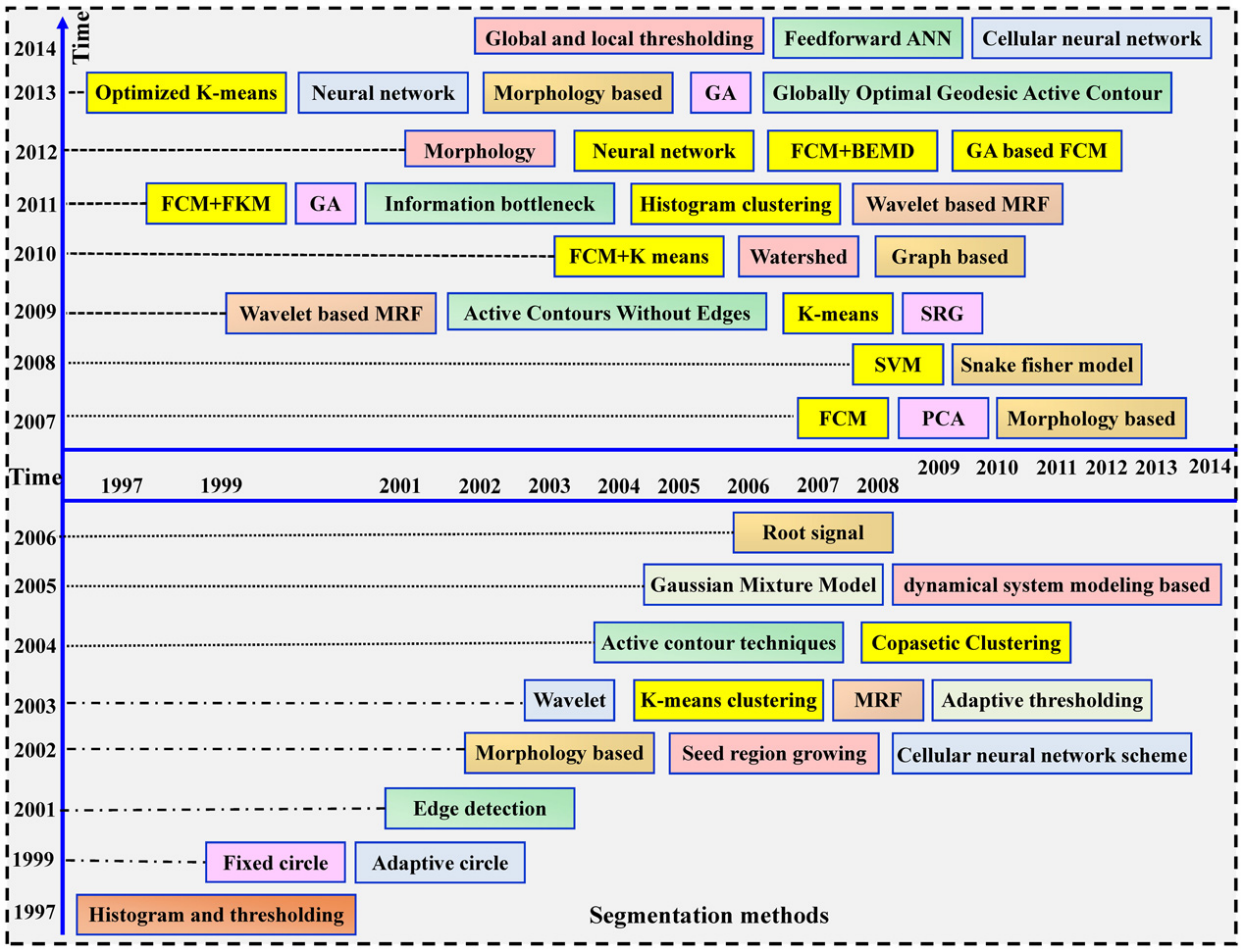
Development history of the segmentation methods.

However, since a spot’s morphology is not always a circle, it is hard for the previously mentioned methods to tackle the real spots accurately and automatically. Therefore, a series of automatic algorithms have been proposed and there is a trend from shape-based segmentation to learning dependent segmentation, as shown in Fig 3.

We can classify these methods into the following categories:

1. Thresholding-based algorithms make use of statistical intensity modeling and find the optimal threshold to segment out the spot [27, 28], but its performance relies on the appropriate choice of background samples.
2. Edge and shape detection-based methods utilize gradients, snakes and active contours to capture the boundary and region information of spot [13–14, 29]. A disadvantage of these methods is that it will give inaccurate results in the presence of noise and artifacts.
3. Morphology-based techniques combine the mathematical morphology operations to realize the spot detection [15, 26, 30–31], yet the structure element is designed humanly and the segmentation effect is strongly dependent on the shape of structure element.
4. Intelligent optimization methods (genetic algorithm or neural network) based algorithms make use of iterative operation to assign pixels into spot or non-spot classes [3, 7, 16–17]. However, it is prone to be affected by noises and requires parameter presetting or network training.
5. Modeling-based algorithms rely on the clustering of pixels’ values to achieve the spot localization [32–33]. One major drawback of these methods is that it ignores the spatial dependencies among adjacent pixels which will lead to an over-segment of the microarray spots.
6. MRF modeling-based method utilizes the neighboring information, along with the intensity information to segment spots [18–19, 34]. However, it requires an initial classification of the pixels and in turn which will affect the final results.
7. Clustering-based algorithms take advantage of K-means, hybrid K-means and fuzzy C-means (FCM) to determine which pixels should belong to the spot or background area [20–21,24–25,35–37]. Nevertheless, these techniques become inaccurate when the spots have poor contrast or they are closer to each other.
8. Other methods utilize pattern recognition or classification to realize the spot segmentation [38–41], yet input of parameters is required for these methods.

All aforementioned algorithms are automatic to some extent, and they also need human intervention to define input parameters or to correct the segmentation results. To summarize the discussions made so far, we can draw the observations that 1) segmentation is an important and challenging problem; 2) image quality needs to be improved for extracting gene expression; 3) a lot of segmentation methods have been proposed with some performances better than others; 4) no single segmentation algorithm can meet the demands of all microarray images, and 5) there has been little progress on developing sufficiently fast, efficient but effective algorithms to segment a microarray image by using up-to-date techniques[7]. In addition, some segmentation algorithms are normally designed to perform well on microarray images acquired by certain types of arrayers and scanners. Thus, it is significant to explore a new method for microarray image segmentation.

## Materials and Methods

**Fig 4:**
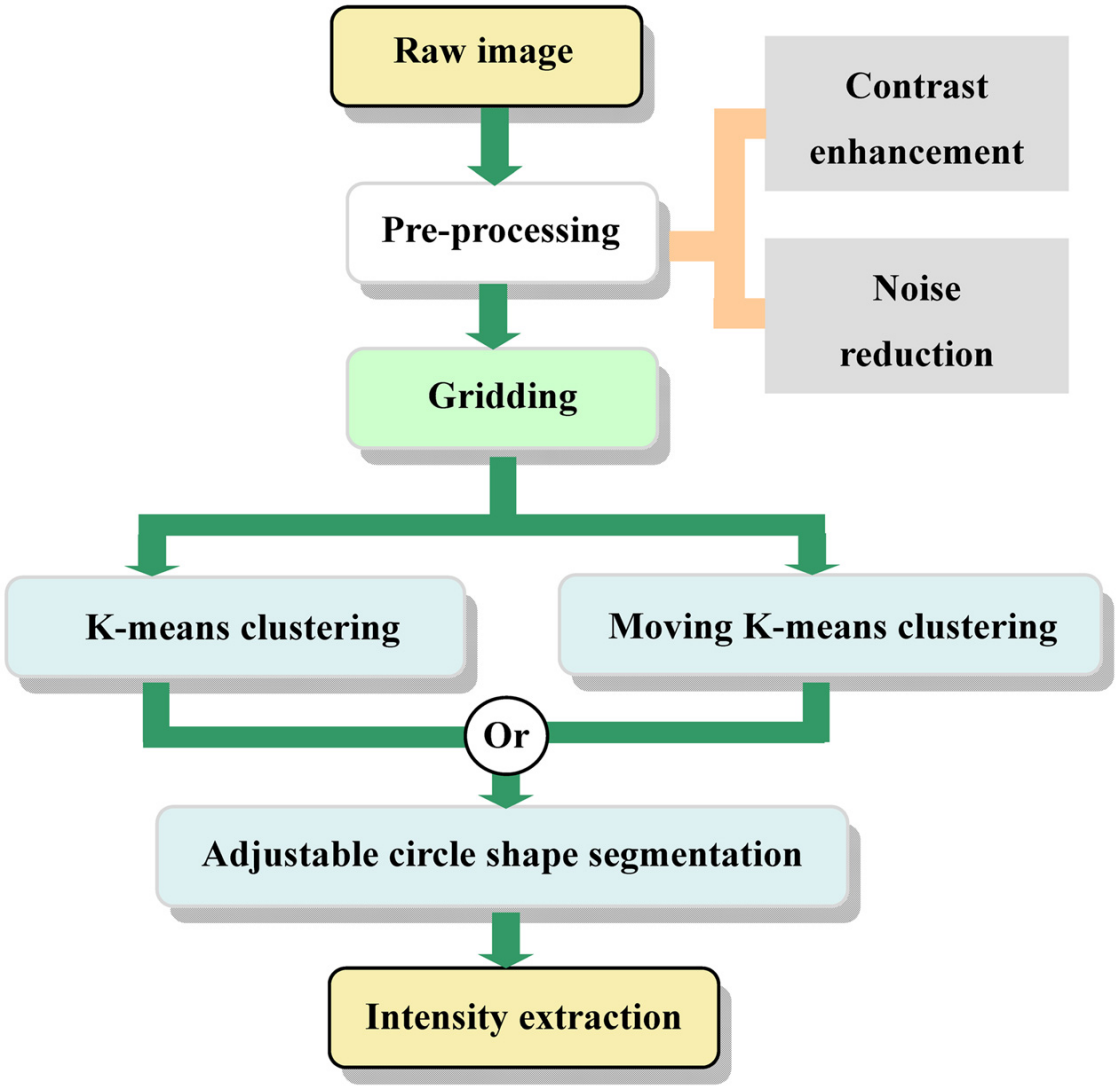
The flowchart of the improved method.

The proposed method mainly consists of five steps: 1) image contrast enhancement, 2) gridding, 3) segmentation, 4) refinement of segmentation, and 5) intensity extraction, as shown in Fig 4.

### Image Quality Enhancement

**Fig 5:**
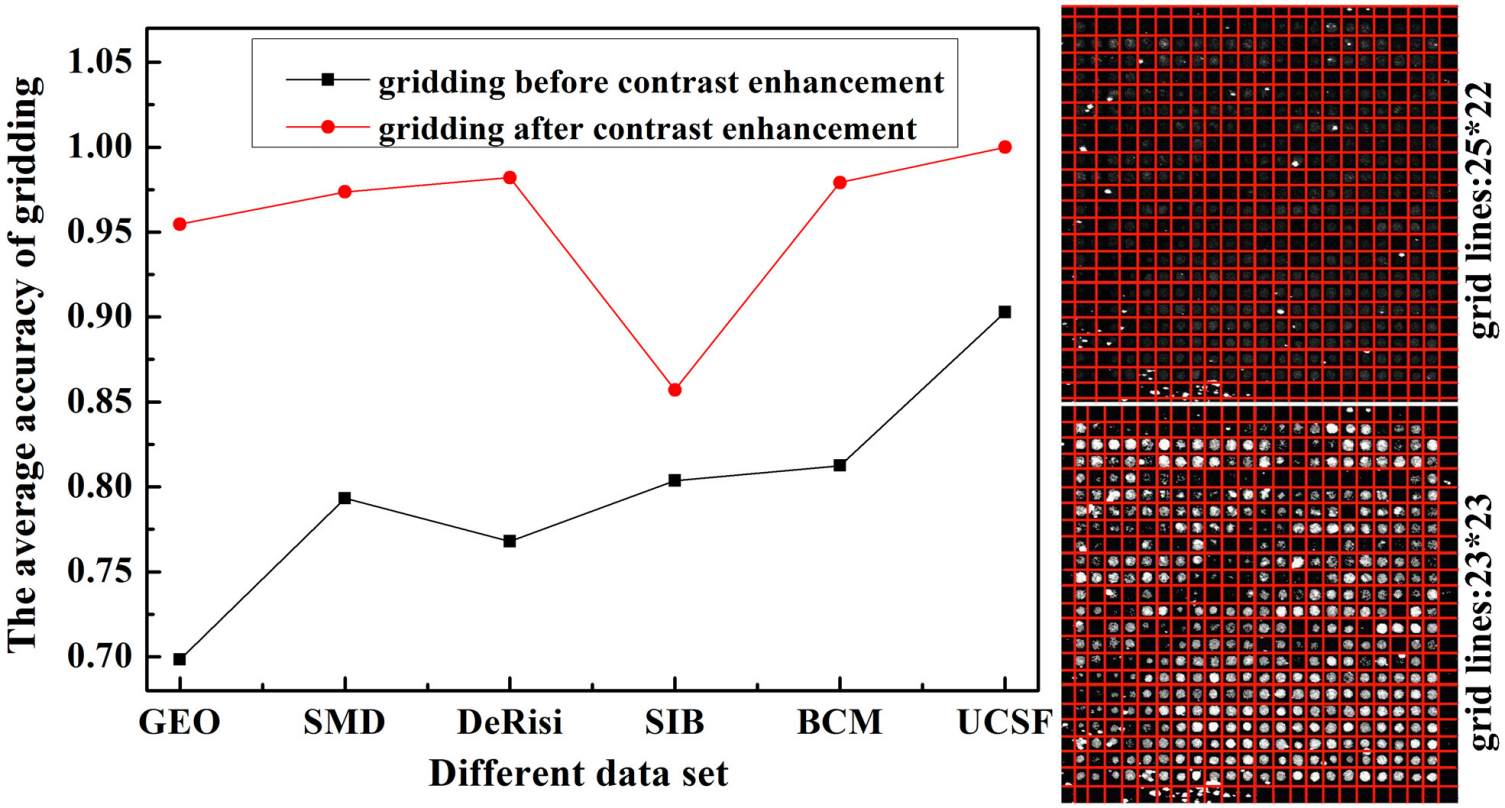
Influence of contrast enhancement on gridding. The right column shows the gridding result before contrast enhancement (top) and result after contrast enhancement (bottom).

We have conducted a comparison experiment on the influence of contrast enhancement on gridding, as shown in Fig 5. Obviously, the gridding accuracy is greatly increased when the image contrast is enhanced compared to those without enhancement. As the example shown in Fig 5, the grid lines 25 on horizontal and 22 on vertical are obtained before contrast enhancement, yet the correct grid lines should be 23 and 23 respectively.

Considering that the low contrast in microarray image between foreground and background makes it difficult to distinguish one from the other, the contrast enhancement is necessary to highlight important features embedded in the microarray image data. Let *f*(*x*,*y*)(*x*∈[1,*w*],*y*∈[1,*h*])represent the gray microarray image,*w* and *h* represent the width and height of the image, respectively. The 2D signal is first transferred into 1D signal *p*. At the same time, in order to only enhance the spots features, the contrast enhanced image *g* can be gained by

$$g(x,y)=\{\begin{array}{cc} f(x,y)*(10000/C) & f(x,y)>k\\ f(x,y) & otherwise \end{array},$$ (1)

in which *C* is the contrast degree estimated automatically by the following equation

$$C=s/{\left[s^{4}/{\left(s^{{}_{2}}\right)}^{2}\right]}^{1/4},\;s={\left[\frac{1}{N}\sum {\left(p-\bar{p}\right)}^{2}\right]}^{1/2}\;s^{4}=\frac{1}{N}\sum {\left(p-\bar{p}\right)}^{4}\;s^{2}=\frac{1}{N}\sum {\left(p-\bar{p}\right)}^{2}$$ (2)

Where $\bar{p}=\frac{1}{N}\sum p$ is the mean value, *s* means a standard deviation, *s* ^2^ represents the mean square error, and *s* ^4^ denotes the four-order moment.

Since spots play a fundamental role in microarray image understanding, one good way to enhance the contrast is to enhance the spots. Therefore, we propose a method to estimate the background pixel value *k* by performing following steps:

1. Randomly select an area *A*_*j*_ by a 10×10 rectangular shape window in an image edge region. For reasonability, choose 3 blocks in each top, left, right and bottom edge region, respectively.
2. Compute the maximum gray value in each area.
3. Adopt the minimum one as background gray value among all maximum gray values.
4. Repeat step 1) to 3) for *m* times to avoid noise effects, here *m* = 10, therefore the background pixel value can be defined as
  $$k=\frac{1}{m}\sum_{i=0}^{m}\underset{i}{\text{min}}\{\underset{j}{\text{max}}A_{j}\}(j\in [1,12])$$ (3)

Finally, a 3×3 median filter is adopted to reduce noises.

### Gridding

**Fig 6:**
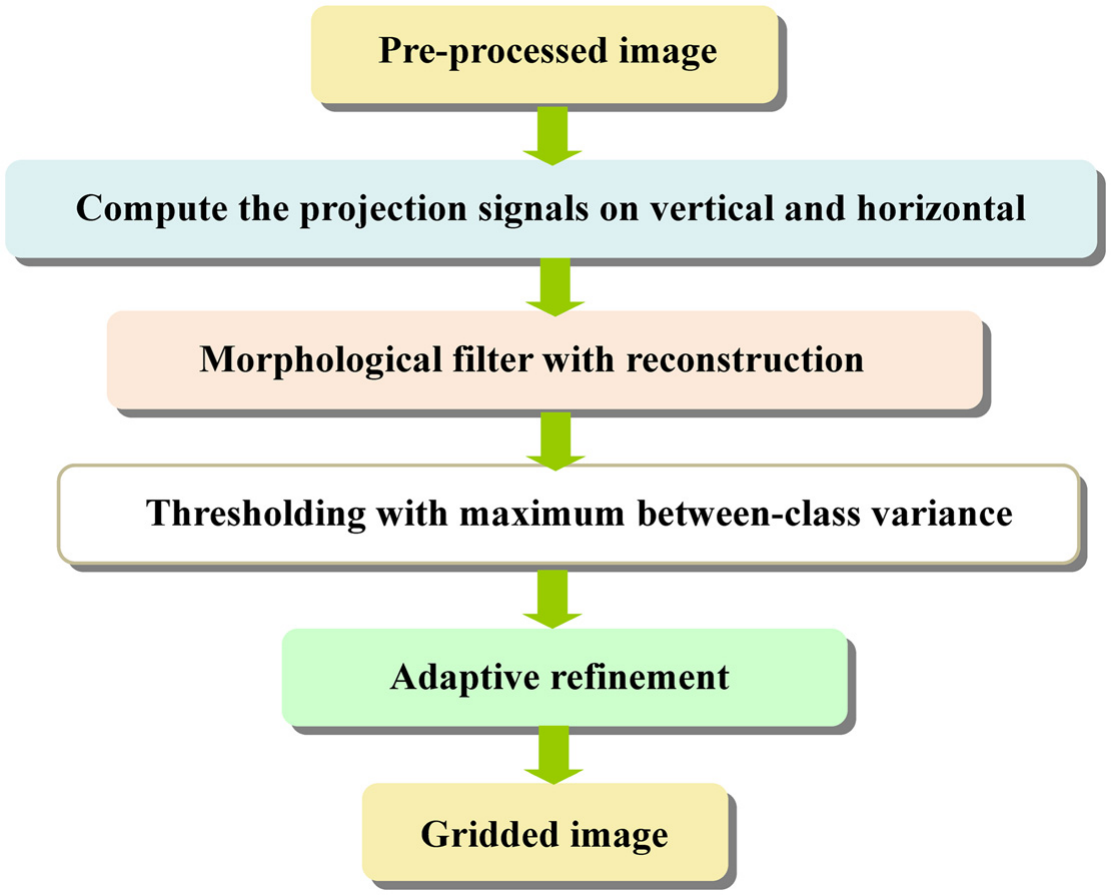
Steps of microarray image gridding.

The main role of gridding is to divide spots into independent areas, and here we adopt the gridding method previously designed by us [42]. The steps of gridding process are shown in Fig 6.

1. First, the contrast enhanced image *g*(*x*,*y*) is used as input for gridding.
2. Then, the horizontal and vertical projection signals are computed by $H(y)=\sum_{x=0}^{w-1}g(x,y)$ and $v(x)=\sum_{y=0}^{h-1}g(x,y)$, respectively.
3. A morphological reconstruction of $H\prime =H-reconstruct\{(H-\bar{H}),H\}$ or $V\prime =V-reconstruct\{(V-\bar{V}),V\}$ is introduced to filter the projection signal, in which $\bar{H}=(\sum H)/\text{h}$ or $\bar{V}=(\sum \text{V})/\text{W}$ is the mean.
4. Next, the maximum between-class variance operation with $d=\frac{\mu_{\delta }{\left(\omega -\mu_{\delta t}\right)}^{2}}{\omega (1-\omega )}$ is developed to look for the optimal threshold.
  - Take the horizontal signal *H*′ as example, let *L* denote the maximum gray value and *n*_*i*_ represent the number of pixels at gray level *i*, its corresponding probability *P*_*i*_ = *n*_*i*_ */h*,*i*∈[1,*L*] and the total mean $\bar{u}=\sum_{i=1}^{L}ip_{i}$ can be computed.
  - Supposing that *t*∈[1,*L*] is a threshold and $\omega =\sum_{i=1}^{t}p_{i}$ describes the occurrence probability of one class divided by the threshold *t*, the image average variance $\mu_{\delta }^{2}=\frac{1}{L}\sum_{i=1}^{L}{\left(i-\bar{u}\right)}^{2}p_{i}$ and the class average variance $\mu_{\delta t}^{2}=\frac{1}{t}\sum_{i=1}^{t}{\left(i-u_{t}\right)}^{2}p_{i}$ can be obtained, in which $u_{t}=\sum_{i=1}^{t}ip_{i}$ denotes the first order cumulative moments of the histogram up to *t* th level.
  - Change the threshold *t* and recalculate the between-class variance until a maximum *d* is gained.
5. Subsequently, the horizontal signal *H*′is transferred into a binary signal according to the threshold *d*.
6. The grid lines coordinate vector *HL* = [*h*_1_,*h*_2_,…,*h*_l+1_],*h*_*i*_ ∈[1,*h*] and the number of spots on each horizontal line *l* can be obtained based on searching for the edge hop. Similarly, by doing all above steps the grid lines coordinate vector *VL* = [*v*_1_,*v*_2_,…,*v*_*s*+1_],*v*_*j*_ ∈[1,*w*] and the number of spots on each vertical column *s* for vertical projection signal *V*′can also be acquired.
7. However, some grid lines may locate at the spot even though the spots number *l* and *s* computed by our proposed method are correct. Therefore, we put forward a refinement step based on the statistical analysis of the obtained grid lines coordinate data and give some heuristic rules [42].
8. Finally, an accurate gridding of the microarray image can be obtained based on the above mentioned steps.

### Segmentation

After gridding, all spots are divided into different parts. Then k-means clustering and moving k-means clustering are executed separately in each spot area. First of all, the feature selection for clustering is crucial. As for each pixel, there are two basic features defined as intensity for the Red channel and Green channel [21]. In addition, the rows of the pixel, columns of the pixel and the Euclidean distance are also introduced for spatial description [20]. Recently, to segment the spot by classification, shape features have also been proposed besides the mean intensity, intensity standard deviation and entropy of intensity features [38]. In this paper, we selected five features as described in Table 1.

**Table 1:** The features used in our improved method.

Type	Edge detection	Description
**Spatial features**	*i*	row of the pixel
	*j*	column of the pixel
	$\Delta =\sqrt{{\left(i-i_{c}\right)}^{2}+{\left(j-j_{c}\right)}^{2}}$	euclidean distance of each pixel to clustering center,*i*_*c*_,*j*_*c*_ are row and column of clustering center
**Intensity features**	*g*_*R*_(*i*,*j*)	intensity of pixel (i,j) for Red channel
	*g*_*G*_(*i*,*j*)	intensity of pixel (i,j) for Green channel

Based on these features, the k-means clustering and the moving k-means clustering can be conducted. To take advantage of both algorithms, the final result is defined by

$$g\prime (x,y)=g_{1}(x,y)||g_{2}(x,y).$$ (4)

To be specifically, the moving k-means clustering is first performed for all spots within their regions and the clustering result *g*_1_(*x*,*y*) can be obtained. Then, count the number of pixels belonging to the foreground *N*_*t*_ and background *N*_*b*_ in each spot area, respectively. If *N*_*t*_<*N*_*b*_ conduct the k-means clustering and obtain another result *g*_2_(*x*,*y*). Next, combine these two results by Eq 4.

The moving k-means clustering method consists of the following steps [4].

1. Extract the spot area according to the gridding result.
2. Select the maximum and the minimum gray value as the original clustering center *c*_1_ and *c*_2_ for class *S*_1_ and *S*_2_, respectively.
3. Initialize parameter *α*_0_ (0<*α*_0_<1/3) and let *α*_*a*_ = *α*_*b*_ = *α*_0_.
4. Compute the Euclidean distance of each pixel *g*_*i*_ to the two clustering center by Δ_*j*_ = ||*g*_*j*_−*c*_*j*_||,*j* = 1,2.
5. If Δ_1_<Δ_2_ it indicates that *g*_*i*_ is closer to class *S*_1_, then merge *g*_*i*_ into the closer class *S*_1_, and vice versa.
6. Update the new clustering center $c_{j}=\frac{1}{N_{j}}\sum_{i\in S_{j}}g_{i}$. *N*_*j*_ represents the pixel number in class *S*_*j*_.
7. Define a new fitness function and compute the fitness for two clustering centers $F(c_{j})=\sum_{i\in c_{j}}{\left(\left|\left|g_{i}-c_{j}\right|\right|\right)}^{2}$.
8. Compare the two fitness values and denotes the higher and lower one as *c*_*h*_,*c*_*l*_.
9. If *F*(*c*_*l*_)<*a*_*a*_ *F*(*c*_*h*_) and there is g_*i*_<*c*_*h*_ for the pixel within *c*_*h*_, then classify it into the class *c*_*l*_. Calculate the new clustering center with $c_{h}=\frac{1}{n_{h}}\sum_{i\in c_{h}}g_{i}$,$c_{h}=\frac{1}{n_{h}}\sum_{i\in c_{h}}g_{i}$.
10. Update *α*_*a*_ with *α*_*a*_ = *α*_*a*_/2 and repeat step 8) to 9) until *F*(*c*_*l*_)≥*a*_*a*_ *F*(*c*_*h*_).
11. Repeat step 4) through 6).
12. Update *α*_*a*_,*α*_*b*_ with *α*_*a*_ = *α*_0_ and *α*_*b*_ = *a*_*b*_/2. Repeats step 7) to 11) until *F*(*c*_*l*_)≥*α*_*b*_ *F*(*c*_*h*_).
13. Finally, the segmented result *g*_1_(*x*,*y*) can be achieved.

The operation of k-means clustering method is similar to the step 1) to 6) of the moving k-means clustering algorithm, so that we also can obtain another segmented result *g*_2_(*x*,*y*).

In addition, the circle shape segmentation will be carried out when *N*_*t*_<0.3(*N*_*t*_+*N*_*b*_), where the diameter of circle is automatically obtained in the gridding step.

### Intensity Extraction

After segmentation, each spot area can be located. Then the intensity extraction operation is conducted on the original raw image *f*(*x*,*y*). The reason for extracting the spot intensity on the raw image, instead of the preprocessed image, is that the preprocess step will result in the image information changing. Especially for our contrast enhancement operation which only enhance the spot gray values with those of background remaining unchanged, as described in Eq 1. Take one sub-grid of image 49 from the SMD data set for example, owing to the contrast enhancement that is automatically processed, the contrast of channel 1 is magnified 24 times and channel 2 15 times. If we use the preprocessed image to extract the intensity, a big error will occur when counting the gray value of the foreground. The following steps are defined to extract the spot intensity:

1. Compute the gray value of the foreground and background $R_{t}=\sum_{i\in t}f_{i}$ and $R_{b}=\sum_{i\in b}f_{i}$, respectively.
2. Obtain the average gray value of the foreground and background by $M_{t}=\frac{R_{t}}{N_{t}}=\frac{1}{N_{t}}\sum_{i\in t}f_{i}$ and $M_{b}=\frac{R_{b}}{N_{b}}=\frac{1}{N_{b}}\sum_{i\in b}f_{i}$.
3. To reduce the effect of background noise, the average gray value of spot is defined as *M*_*e*_ = *M*_*t*_−*M*_*b*_.
4. Repeat step 1) to 3) and compute the average gray value for each microarray image channel, then the spot intensity value can finally be determined by
  $$value=\text{log}(M_{e1}/M_{e2}).$$ (5)

## Results and Discussion

### Experiment Setting

To verify the validity of the proposed method, we implemented our programs in Matlab R2010a and ran them on the Intel-based workstation with Windows XP OS. We selected six data sets, namely 1) SMD (http://smd.stanford.edu), 2) GEO (http://www.ncbi.nlm.nih.gov/geo/), 3) BCM (http://xmushaogf.blog.163.com/), 4) DeRisi (http://www.bio.davidson.edu/projects/magic/magic.html), 5) UCSF (http://cancer.ucsf.edu/cores/arraysampledata), and 6) SIB (http://www.isrec.isb-sib.ch/). All the blocks were stored in TIFF files with 16-bit gray level depth. The specifications of each data set are shown in Table 2[42].

**Table 2:** Data set details for image used in this paper.

Data set	GEO	BCM	SMD	UCSF	SIB	DeRisi
Name	Gene Expression Omnibus	Bachelor College of Medicine	Stanford Microarray Database	University of California, San Francisco	Institute for Experiment Cancer Research	Joe DeRisi Individual
No. of sub-grid	464	484	528	72	56	56
Spot layout	13×14	22×22	14×18	14×15	5×7 to 7×7	40×40
Spot resolution	12×12	25×25	18×18	8×8	18×18	8×8
Image Resolution	451×391	943×949	460×451	218×209	366×350	463×455

In addition, the proposed method is compared with other four algorithms, 1) the edge detection method [1], 2) the thresholding method [35], 3) the k-means clustering method [20], and 4) the moving k-means clustering method [25]. Especially, all the images used for comparison experiments are processed by the same contrast enhancement algorithm proposed in this paper and the maximum between-class variance gridding method proposed by the authors [42].

### Performance

**Fig 7:**
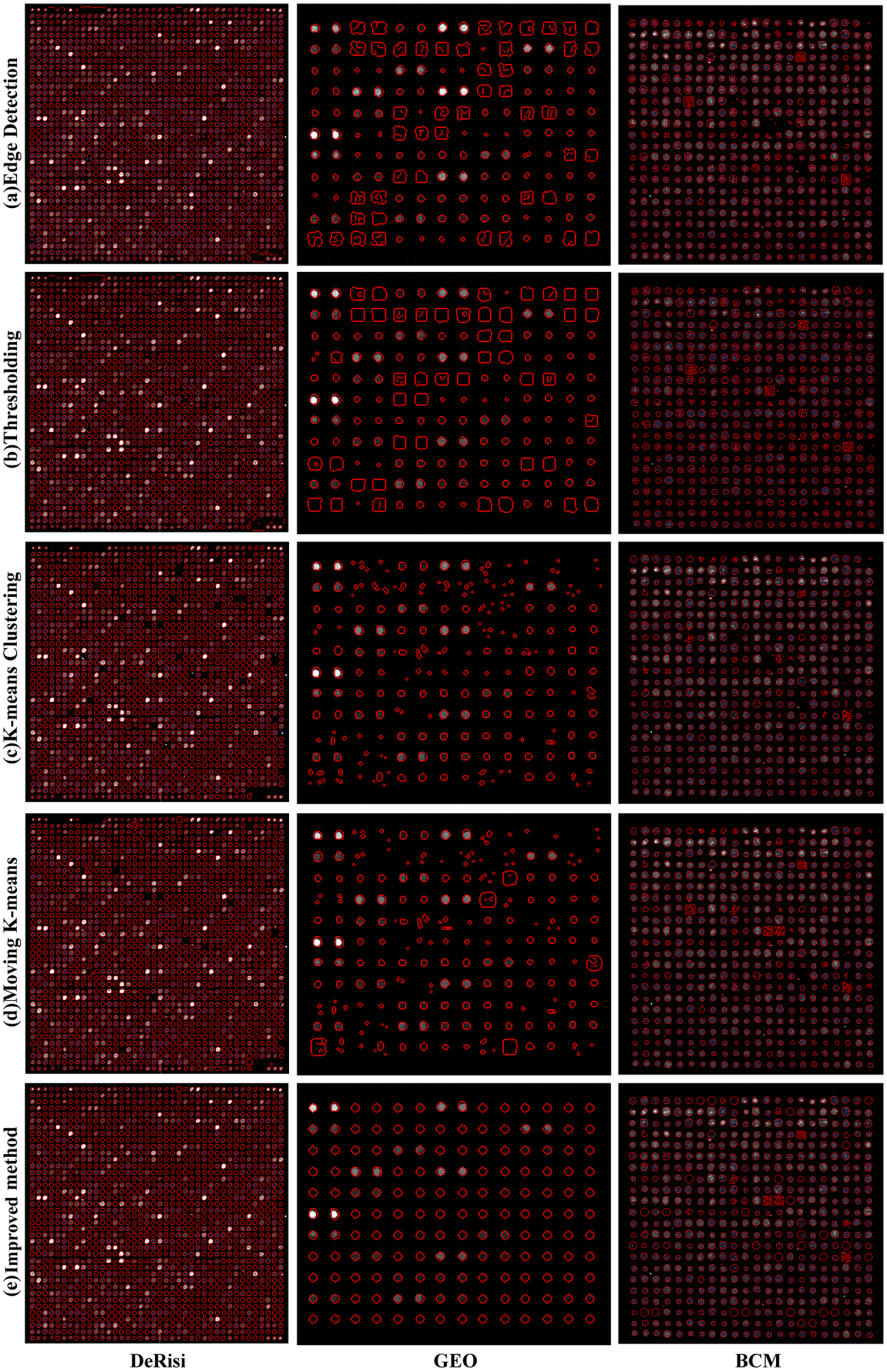
Comparison of five segmentation methods on three data sets.

Fig 7 exhibits the segmentation results of five different methods on three data sets. It can be seen from this figure that a lot of spots are detected as the whole spot area boundary instead of its real shape in the edge detection method. Similar results are found in the thresholding method. In terms of k-means clustering method and the moving k-means algorithm, the former can segment the spot close to its real edge, while the latter is able to detect the spot with lower contrast. However, there still exists the situation of the discontinuous area and irregular shapes in these two clustering methods. Moreover, all the methods perform better on the DeRisi data set owing to its sub-grid having a dense spot layout. All in all, although the proposed method performs best, some spots still can’t be segmented accurately.

**Fig 8:**
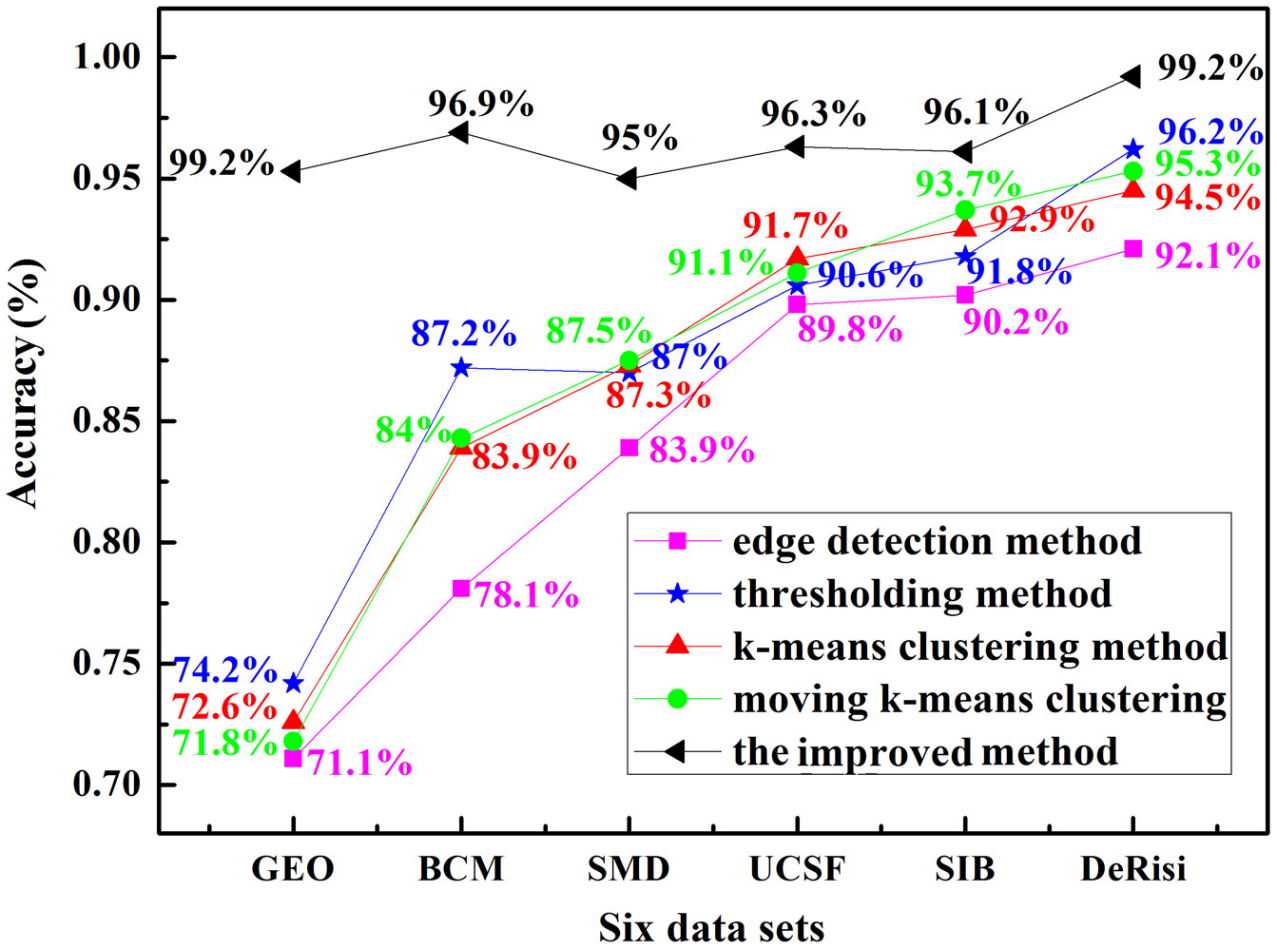
The average segmentation accuracy of five methods on six data sets.

Fig 8 shows the average segmentation accuracy of these five methods on six data sets. The accuracy is defined as the correct segmentation number of spots/total number of test spots. Clearly, the imprved method has remarkably higher accuracy than the other four methods. Among these algorithms, the edge detection method gains the lowest accuracy on all situations, while the improved method is best performed at all times. In addition, the moving k-means clustering algorithm always performed better than the k-means clustering method. What’s more, there is a similar trend on all data sets, which is all the algorithms gain a highest accuracy on the DeRisi data set and lowest on GEO data set. The reason for such lower segmentation accuracy of the other four methods on the GEO data set is that most sub-grids have low contrast. While the sub-grids in the DeRisi data set have the higher contrast. Particularly, our algorithm improved the accuracy for almost 20% after contrast enhancement. All above analysis proved that the contrast enhancement for microarray image was crucial.

### Segmentation in the Presence of Noise

**Fig 9:**
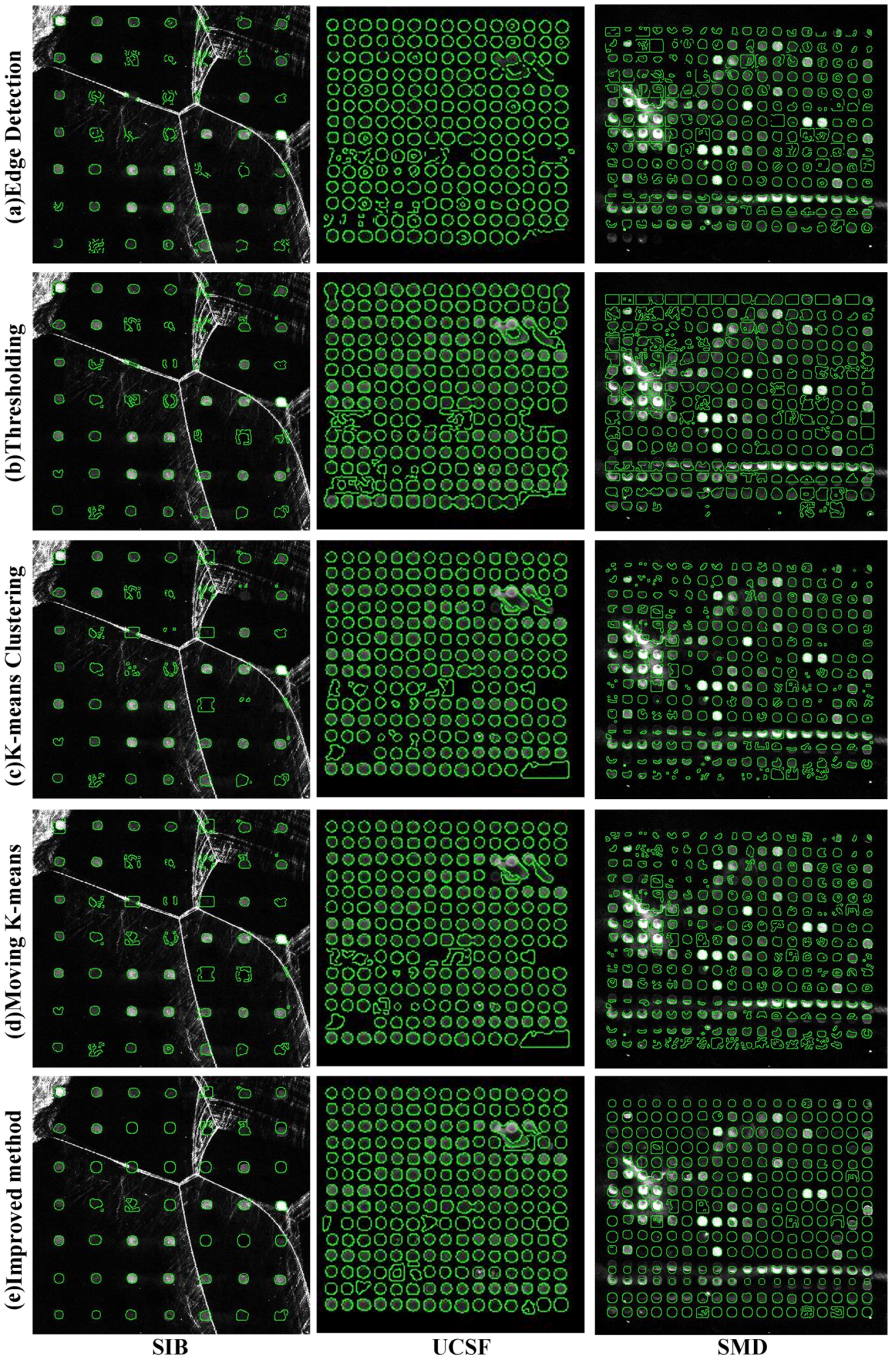
Segmentation examples of five methods in the presence of noises.

To further verify the effect of the improved method we selected some sub-grids at the presence of noises from six data sets. Fig 9 shows the segmentation examples of five methods on three data sets. It can be seen that the edge detection method and the thresholding method classify the noises into spots. What’s more, they can’t recognize the spots those have much lower contrast. For the k-means clustering method and the moving k-means clustering method, they only locate partial of the spots owing to the effect of noises. The improved method proposed performs better than all the other four algorithms. However, it will be a little bit affected by noises so that some spots are located with extra boundary.

**Fig 10:**
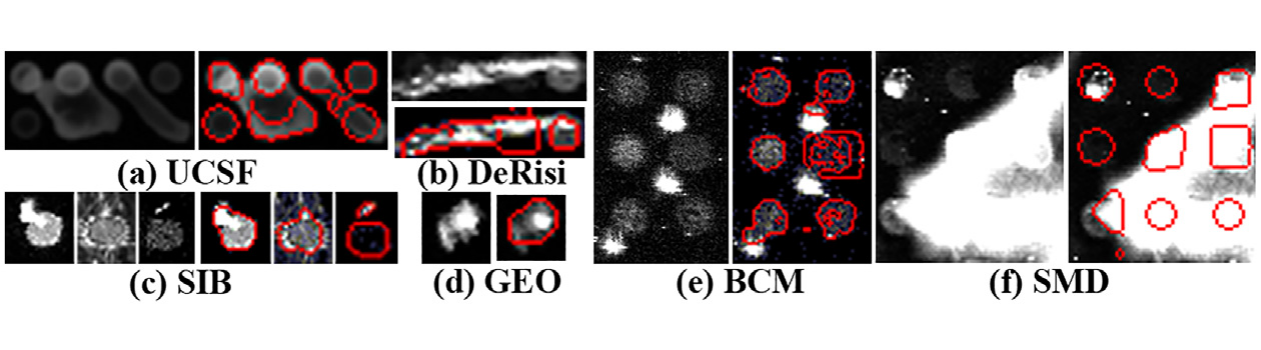
Segmentation examples fail to obtain boundary of spots.

Furthermore, Fig 10 exhibits some examples that our improved method fails to obtain the boundaries of spots. For example, a crescent moon shape spot is obtained as shown in Fig 10a owing to the noises in that region have similar fluorescent with that of spot. Due to noises have higher gray values than spots so they are classified into spots by mistake as shown in Fig 10b,10c,10d,and 10e. In addition, a square shape spot is segmented in Fig 10f because that area is fulfilled with noises.

Table 3 exhibits the segmentation accuracy when noise appears. The accuracy of detected spots are divided into incorrectly, marginally and correctly [43], here the marginally, which is different from the definition for gridding, means the spot edge detection is correct but with some holes in it. The segmentation accuracy on the SIB data set dropped a lot owing to its sparse distribution of spots, which makes the algorithm prone to be affected by noise. In addition, owing to the uneven distribution of spot gray values, there are some holes in the detected spots on different data sets.

**Table 3:** The average segmentation accuracy of five methods on six data sets in the presence of noises.

Data sets		Accuracy (%)					
		GEO	BCM	SMD	UCSF	SIB	DeRisi
**Edge detection**	correctly	56.7	73.1	39.6	84.72	39.6	96.0
	marginally	0.0	4.9	0.08	0.08	1.2	0.0
	incorrectly	43.3	22.0	59.6	15.2	59.2	4.0
**Thresholding**	correctly	55.5	80.9	53.5	83.8	44.9	92.6
	marginally	0.0	8.3	0.06	0.0	0.0	0.0
	incorrectly	44.5	10.8	45.9	16.2	55.1	7.4
**K-means clustering**	correctly	70.3	81.6	61.1	86.7	53.1	90.9
	marginally	0.0	0.0	0.04	0.01	0.0	0.0
	incorrectly	29.7	18.4	38.5	13.3	46.9	9.1
**Moving k-means clustering**	correctly	70.7	82.9	61.8	86.08	62.9	93.1
	marginally	0.0	0.0	0.05	0.02	0.04	0.0
	incorrectly	29.3	17.1	37.7	12.9	36.7	6.9
**The improved method**	correctly	92.3	94.5	90.89	95.19	85.692	97.8
	marginally	0.0	0.0	0.01	0.01	0.008	0.0
	incorrectly	7.7	5.5	9.1	4.8	14.3	2.2

### Segmentation of Spots with Special Shapes

**Fig 11:**
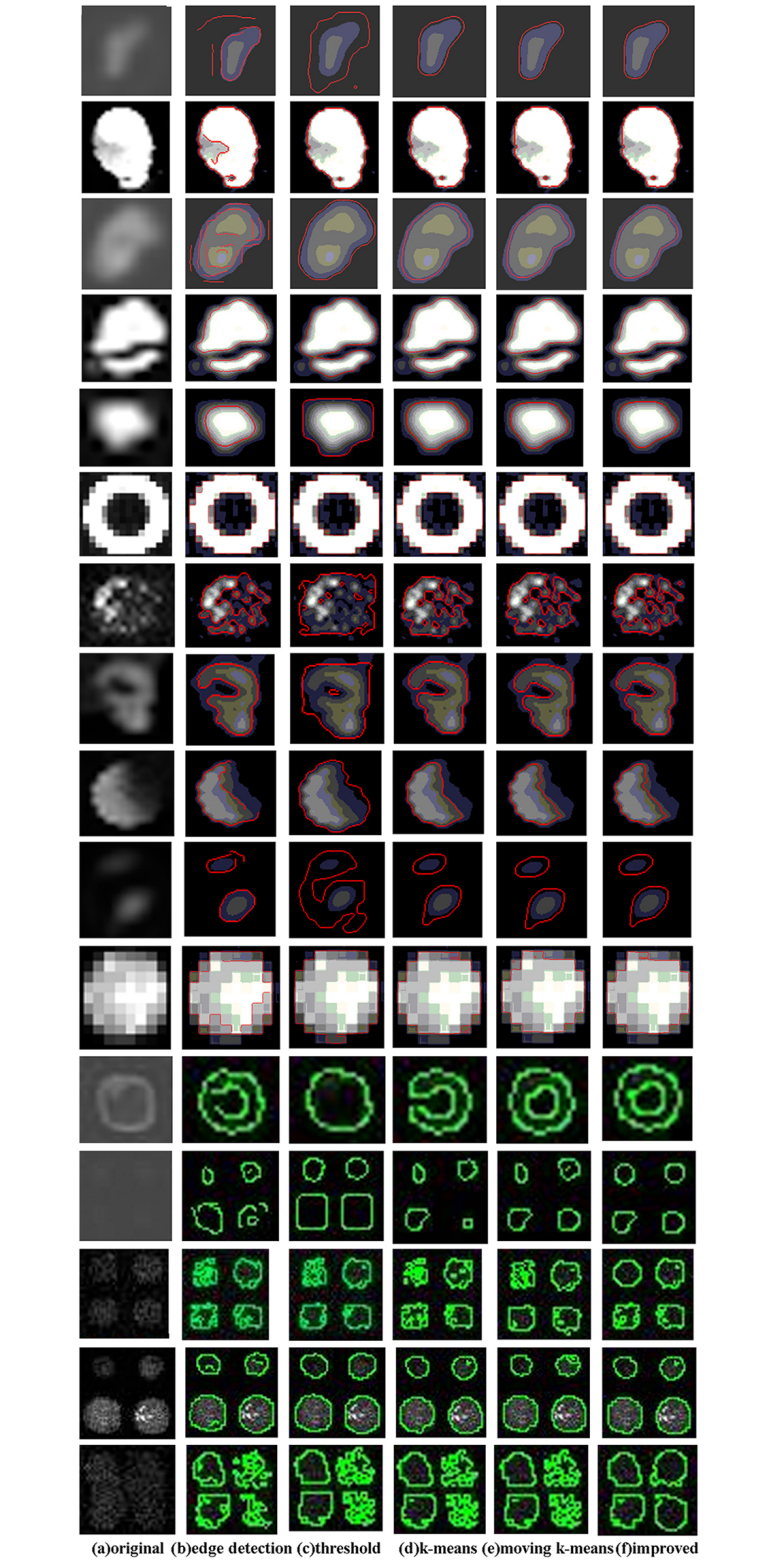
Segmentation examples on spots have different shapes.

Fig 11 presents the segmented results in the case of spots with different sizes, shapes and contrast. Clearly, a much better segmentation outcome can be obtained by using clustering. The segmented results of the improved method are robust. No matter if the spots are adherent, or edge blurry, it can always segment the spots more accurately and the segmented edge is close to the spot’s real margin.

What’s more, to perform the quantitative analysis, we selected 10 similar spots from each data set. At the same time, the number of pixels clustered to spots *m*_*i*_ is counted and the root mean square error $RMSE={\left(\frac{1}{10}\sum_{i=1}^{10}{\left(m_{i}-m_{i}^{\prime }\right)}^{2}\right)}^{1/2}$ is also calculated, where $m_{i}^{\prime }$ represents the actual number of spots counted manually.

**Fig 12:**
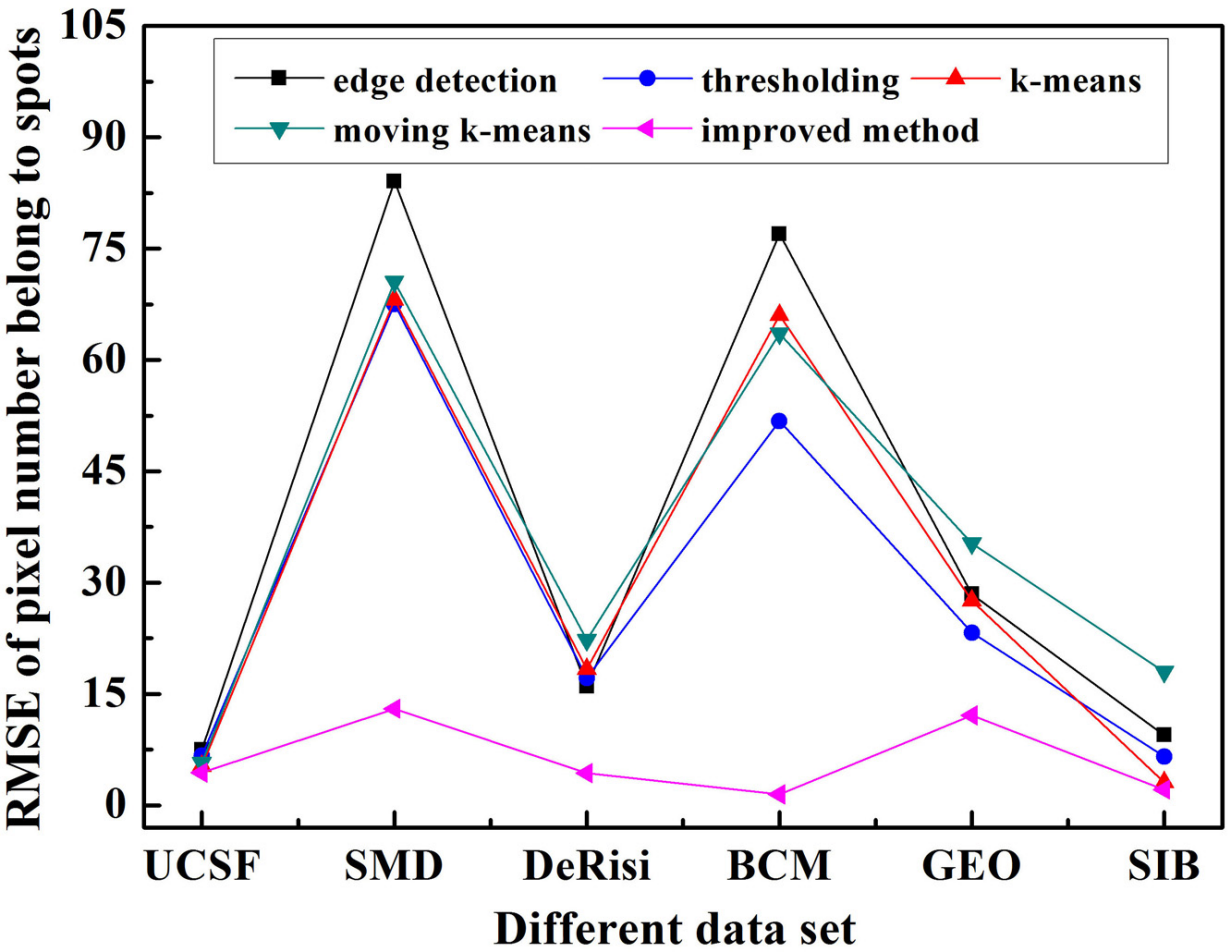
RMSE of pixel numbers using five methods on six different data sets.

Fig 12 exhibits the RMSE values of different methods on six data sets. Among all of the methods, the improved method performed better on all data sets, namely its spot region is similar with the real ones, while the edge detection method gives a poor outcome all the time. For data sets, all methods gain worse results on SMD, GEO and BCM data sets, which can be attributed to 1) lower contrast of spots, 2) irregular shapes of spots, and 3) noise affect. Although there are only 10 spots used, it is consistent with the accuracy shown in Fig 8.

The average number of pixels which belong to 10 spots is presented in Table 4. It can be observed that the result obtained by the improved method is closer to the standard ones. Of course, there are some errors because the standard number of spots is counted manually, and the randomly selected 10 spots can’t represent the whole image. However, the statistical result reveals a tendency in accordance with the former analysis.

**Table 4:** Average number of pixels clustered as spots.

Average pixels belong to spots	Standard number	Edge detection	Thresholdingmethod	K-means	Moving K-means	Improved method
GEO	55.6	31	44.6	30.9	42.9	57
BCM	160.6	106	125	111.1	109.5	160
SMD	136.1	108.1	79.1	81.1	73.2	130.5
UCSF	71.7	78.4	77	71.9	69.3	71.3
SIB	165.8	172.3	165	164.2	153.2	164.9
DeRisi	55.4	57.7	43.9	43.9	40.3	54.8

### Intensity Extraction

**Fig 13:**
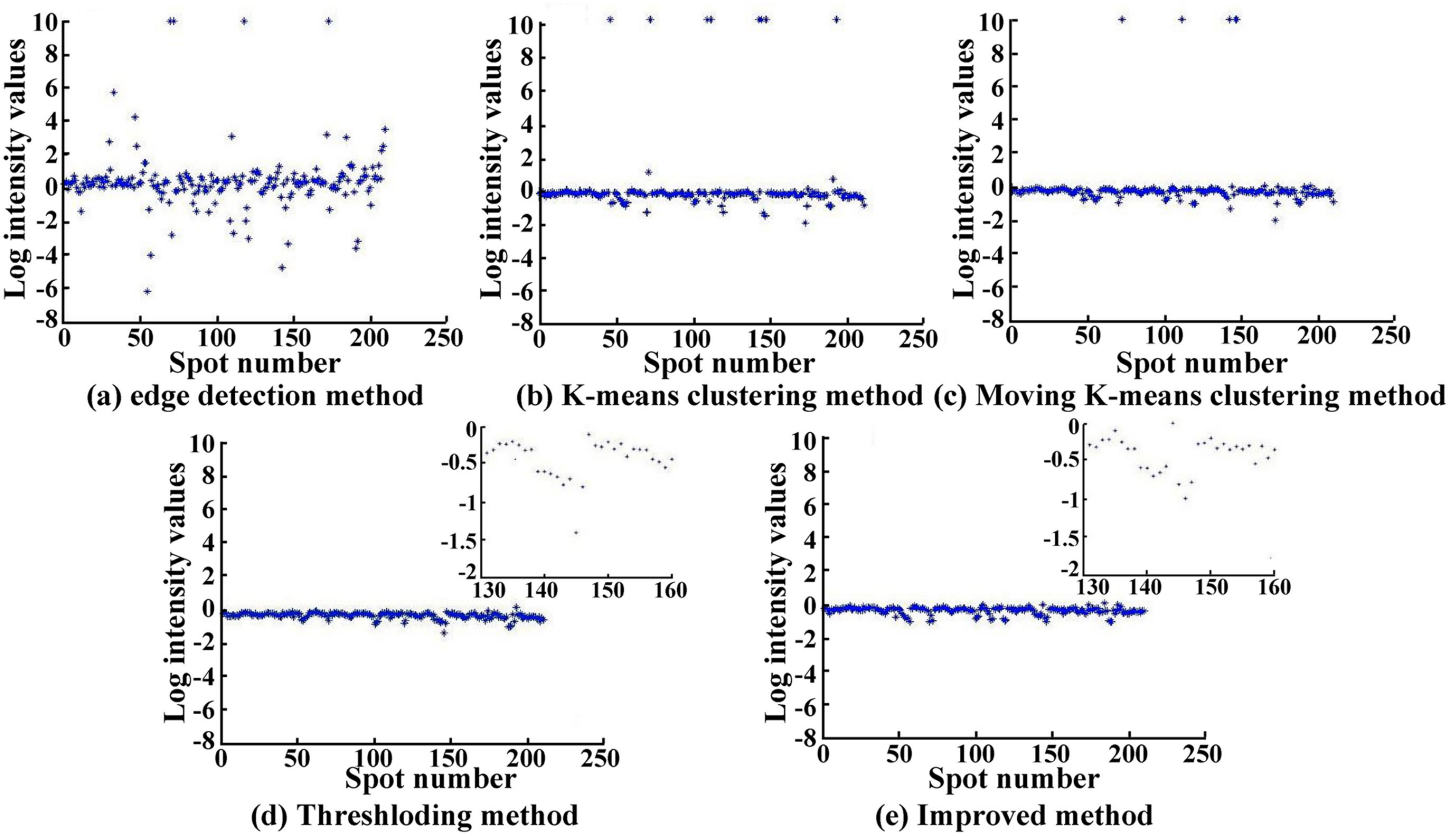
Comparison of log intensity values for five methods, the sub-grid is selected from UCSF data set.

To further analyze the validity of different methods, we plot the intensity distribution for one sub-grid drawn from the UCSF data set, as shown in Fig 13, where the intensity for each spot is calculated by Eq 4. Fig 13 reveals that the improved method obtains a best gene expression outcome with less noise because this method located the spot near to its edge. However, the edge detection method exhibits the worst result with lots of unstable intensity values owing to it divides the background pixels into spots by mistake or vice versa. Meanwhile, the thresholding algorithm also displays the second best stability because it segments the spots with a continuous edge.

In addition, to describe the degree that spot intensity deviates from zero, we calculated the intensity MSE for five methods on six data sets, as shown in Table 5. The improved method possessed the lowest MSE value, meaning that all the intensities are distributed near the region of zero. Because the image in BCM data set contains a large number of noises, there is a higher MSE value.

**Table 5:** The average log intensity values gained by five methods on six data sets.

MSE of Intensity	Edge detection	Thresholding method	K-means	Moving K-means	Improved method
GEO	3.819	1.105	2.563	2.388	0.945
BCM	7.177	2.339	2.936	2.817	2.025
SMD	1.774	0.146	0.391	0.383	0.285
UCSF	2.168	0.109	2.014	1.282	0.095
SIB	5.288	0.846	0.757	0.735	0.788
DeRisi	0.839	0.293	0.460	0.410	0.428

### Computational Efficiency

**Fig 14:**
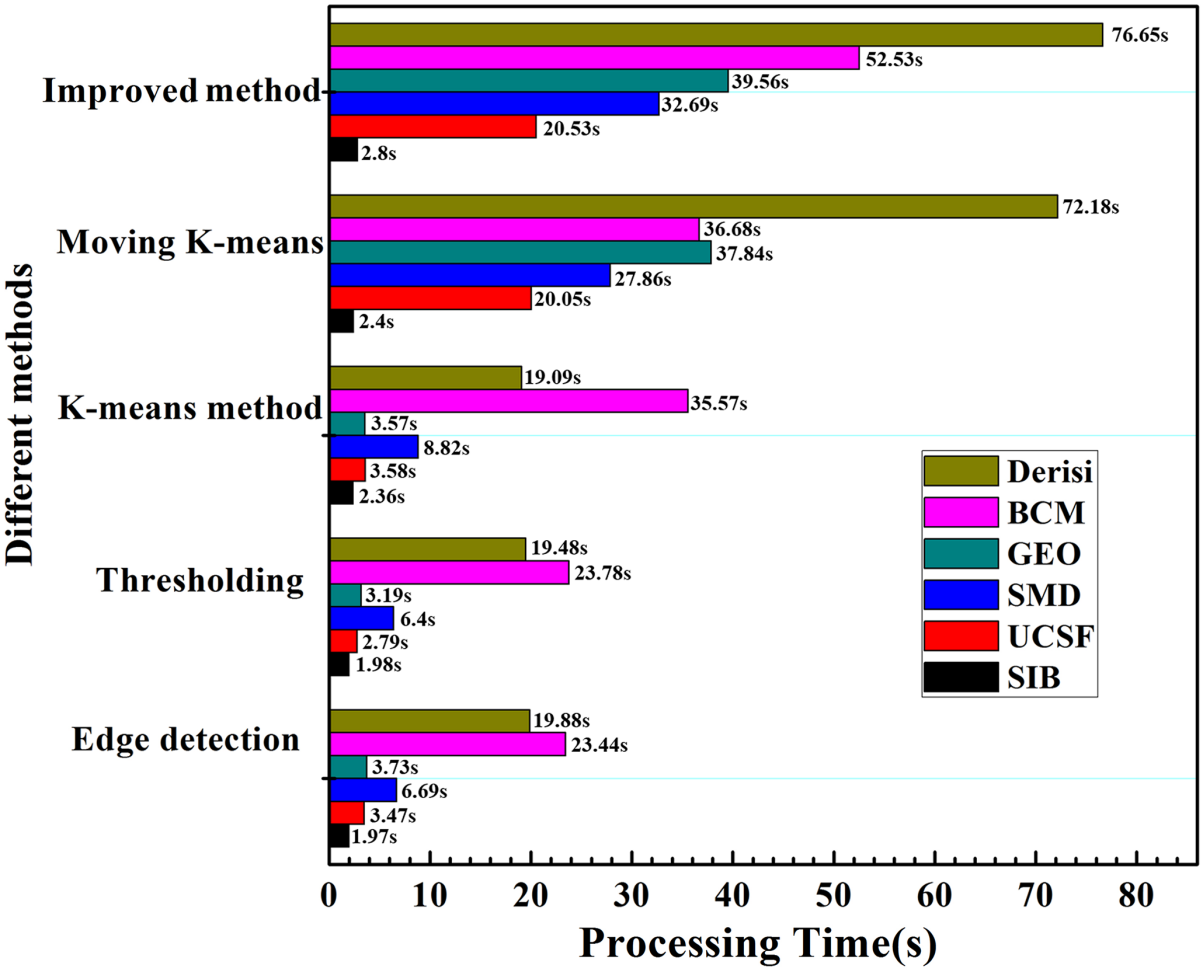
The average processing time (in seconds) of five segmentation methods on six data sets.

Fig 14 exhibits the average time consumption for the whole process of five methods on six different data sets. This processing time starts from sub-grids input to the intensity extraction. There is a different time consumption trend on different data sets owing to the various resolution and spots number of microarray images. Obviously, less processing time is needed for the edge detection method. In contrast, the improved method and the moving k-means clustering algorithm require much more processing time which might be ten times that of the edge detection approach. What’s more, all the methods spend less time on the SIB data set owing to there is only 35 spots in each sub-grid. However, there are 440 and 1,600 spots in each sub-grid drawn from the BCM and DeRisi data sets, respectively, so they require more run-time. Of course, the processing times for all methods are longer because the experiments are done on the Matlab platform. The time consummation will be remarkably decreased if the algorithm is compiled by C language. In addition, the improved method only combines results of the k-means and the moving k-means clustering methods simply with “or” operation. We can design a new scheme to evaluate the spot quality and select the appropriate method to segment it, such as some spots segmented by k-means clustering and others by moving k-means clustering, so that the current processing by these two methods simultaneously can be replaced. Certainly, the dealing time will also decline greatly.

## Conclusions

Microarray technology has been widely applied in drug design, environmental health research, clinical diagnosis and treatment, and in cancer detection. Image processing is a key step of this technology. What’s more, the segmentation step is critical in the processing of microarray image, and the segment accuracy and consistency is also significant. However, owing to the presence of noise, artifacts and laser reflection during microarray experiment procedure, the real microarray image quality varies in spot size, shape, contrast and quantity of missing spots. These dynamics of quality will exert a great challenge to segmentation. In recent years, clustering based approaches such as k-means, fuzzy c-means, and moving k-means, are frequently used in bioinformatics and show better performance than the shape based segmentation ones. However, the conventional clustering based methods tend to face unsatisfactory result when image quality is poor. Therefore, in this paper we proposed an improved method by combining the k-means and the moving k-means clustering methods. Specifically, an automatic contrast enhancement algorithm is used in the pre-processing step to improve the image quality.

The segmentation ability of edge detection, thresholding, k-means clustering, moving k-means clustering and the improved method has been made on six different data sets. Experiment results show that our improved method provides better results than the other four algorithms. It will be more suitable for microarray image segmentation with better performance.

However, it is more time-consuming for about 5 times than that of k-means clustering algorithm. Therefore, optimization of the improved algorithm with less running time may become our forthcoming work, and the GPU-based computing structure for microarray image segmentation will be considered.

## References

[1] Harikiran J, Avinash B, Lakshmi DRPV, Kipankumar DRR. Automatic gridding method for microarray images. Journal of Theoretical and Applied Information Technology. 2014; 65(1):235–41.

[2] Kavitha MG, Kumar DSS. Comparison of clustering techniques for microarray image segmentation. International Journal of Scientific & Engineering Research. 2013; 4 (9):46–50.

[3] Sreedevi A, Jangamshetti DS. Extraction of spots in DNA microarrays using genetic algorithm. An International Journal of Signal & Image Processing. 2013; 4(6):83–93.

[4] Elbs M, Hulko M, Frauenfeld J, Fischer R, Brock R. Multivalence and spot heterogeneity in microarray-based measurement of binding constants. Analytical & Bioanalytical Chemistry. 2007; 387(6): 2017–25. 17260137 10.1007/s00216-006-1098-6

[5] Bajcsy P. An Overview of DNA microarray grid alignment and foreground separation approaches. EURASIP Journal on Applied Signal Processing. 2006:1–13.16758000

[6] Tian MX, Qu J, Han XG, Zhang M, Ding C, Ding JB, et al Microarray-based identification of differentially expressed genes in intracellular brucella abortus within RAW264.7 Cells. PLOS ONE. 2013; 8(8):1–9. 10.1371/journal.pone.0067014 PMC3737221 23950864

[7] Wang Z, Zineddin B, Liang J, Zeng N, Li Y, Du M, et al A novel neural network approach to cDNA microarray image segmentation. Computer Methods Programs Biomedicine. 2013; 111(1):189–98. 10.1016/j.cmpb.2013.03.013 23669179

[8] Katsigiannis S, Zacharia E, Maroulis D. Enhancing the performance of a microarray gridding algorithm via GPU computing techniques 13th IEEE International Conference on BioInformatics and BioEngineering; 2013 11 10–13; Chania, Greece; IEEE; 2013; p.1–4.

[9] Bajcsy P. An Overview of DNA microarray image requirements for automated processing. IEEE Computer Society Conference on Computer Vision and Pattern Recognition. 2005; 147–52.

[10] Zacharia E, Maroulis D. 3-D Spot modeling for automatic segmentation of cDNA microarray images. IEEE Transactions on Nanobioscience. 2010; 9(3):181–92. 10.1109/TNB.2010.2050900 20519160

[11] Lukac R, Plataniotis KN. cDNA microarray image segmentation using root signals. International Journal of Imaging Systems and Technology. 2006; 16(2):51–64.

[12] Wang XH, Istepian RSH, Song YH. Microarray image enhancement using stationary wavelet transform. IEEE Trans Nanobiosci. 2003; 2: 184–9. 10.1109/tnb.2003.816225 15376907

[13] Kim JH, Kim HY, Lee YS. A novel method using edge detection for signal extraction form cDNA micro array image analysis. Exp. Mol. Med. 2001; 33(2):83–8. 11460886 10.1038/emm.2001.15

[14] Ho J, Hwang WL. Automatic microarray spot segmentation using a snake-fisher model. IEEE Transactions on Medical Imaging. 2008; 27(6): 847–57. 10.1109/TMI.2008.915697 18541491

[15] Jr RH, Barrera J, Hashimoto RF, Dantas DO, Esteves GH. Segmentation of microarray images by mathematical morphology. real-time imaging. 2002; 8(6): 491–505.

[16] Zacharia E, Maroulis D. A spot modeling evolutionary algorithm for segmenting microarray images(Chapter 24). Evolutionary Algorithms. InTech Eisuke Kita; 2011; 459–80.

[17] Farouk RM, Badr EM, SayedElahl MA. Recognition of cDNA microarray image using feedforward artificial Neural Network. International Journal of Artificial Intelligence & Applications. 2014; 5(5):21–31.

[18] Demirkaya O, Asyali MH, Shoukri MM, Abu-Khabar KS. Segmentation of microarray cDNA spots using MRF-based method. Proceedings of the 25th Annual International Conference of the IEEE Engineering in Medicine and Biology Society; 2003 9 17–21; 1:674–7.

[19] Athanasiadisa E, Cavourasb D, Kostopoulosb S, Glotsosb D, Kalatzisb I, Nikiforidis G. A wavelet-based markov random field segmentation model in segmenting microarray experiments. Computer Methods and Programs in Biomedicine. 2011; 104(3):307–15. 10.1016/j.cmpb.2011.03.007 21531035

[20] Wu SH, Yan H. Microarray image processing based on clustering and morphological analysis. Proceedings of the First Asia-Pacific bioinformatics conference on Bioinformatics. 2003; 19:111–8.

[21] Giannakeas N, Fotiadis DI. An automated method for gridding and segmentation of cDNA microarray images. Computerized Medical Imaging and Graphics. 2009; 33 (1):40–9. 10.1016/j.compmedimag.2008.10.003 19046850

[22] Yeganeh SH, Habibi J, Abolhassani H, Shirali-Shahreza S. A novel clustering algorithm based on Circlusters to find arbitrary shaped clusters. International Conference on Computer and Electrical Engineering; 2008 12 20–22; Phuket; p.619–24.

[23] Ng MK, Wong JC. Clustering categorical data sets using tabu search techniques. Pattern Recognition. 2002; 35 (12) 2783–90.

[24] Harikiran J, RamaKrishna D, Phanendra ML, Lakshmi PV, Kiran R. Fuzzy c-means with bi-dimensional empirical mode Decomposition for segmentation of microarray image. International Journal of Computer Science Issues. 2012; 9(3):316–21.

[25] Maguluri LP, Rajapanthula K, Srinivasu PN. A comparative analysis of clustering based Segmentation Algorithms in Microarray Images. International Journal of Emerging Science and Engineering. 2013; 1(5):27–32.

[26] Kadam AB, Manza RR, Kale KV. A novel approach for microarray spot segmentation & detection using four shaped mathematical morphology. Advances in Computational Research. 2012; 4(2):130–3.

[27] Liewa AWC, Hong YN, Yang MS. Robust adaptive spot segmentation ofDNA microarray images. Pattern Recognition. 2003; 36 (5): 1251–4.

[28] Wanga ZD, Zineddin B, Liangc J, Zeng N, Li Y, Du M, et al cDNA microarray adaptive segmentation. Neurocomputing. 2014; 142(22): 408–18.

[29] Ni SH, Wang P, Paun M, Dai WZ, Paun A. Spotted cDNA microarray image segmentation using ACWE. Romanian Journal of Information Science and Technology. 2009; 12(2):249–63.

[30] Angulo J. Polar modeling and segmentation of genomic microarray spots using mathematical morphology. Image Analtsis & Stereology 2008; 27(2):107–24.

[31] Manjunath SS, Shreenidhi BS, Nagaraja J, Pradeep BS. Morphological Spot detection and analysis for microarray images. International Journal of Innovative Technology and Exploring Engineering. 2013; 2(5):189–93.

[32] Li QH, Fraley C, Bumgarner RE, Yeung KY, Raftery AE. Donuts, scratches and blanks: robust model-based segmentation of microarray images. Bioinformatics. 2005; 21(12):2875–82. 15845656 10.1093/bioinformatics/bti447

[33] Blekas K, Galatsanos NP, Likas A, Lagaris IE. Mixture model analysis of DNA microarray images. IEEE Transactions on Medical Imaging. 2005; 24(7): 901–9. 16011320 10.1109/tmi.2005.848358

[34] Athanasiadis EI, Cavouras DA, Glotsos DT, Veorgiadis GP (2009) Segmentation of complementary DNA microarray images by wavelet-based markov random field mode. IEEE Transactions on Information Technology in Biomedicine l 13(6):1068–1074. 10.1109/TITB.2009.2032332 19783509

[35] Rajkumar P, Vennila Ila, Nirmalakumari K (2013)An intelligent segmentation algorithm for microarray image processing. International Journal on Computer Science and Engineering 5 (6):528–537.

[36] Uslan V, Bucak IÖ. Clustering-based spot segmentation of cDNA microarray images. Proceedings of the International Conference of IEEE Engineering in Medicine and Biology Society. 2010; 1828–31. 10.1109/IEMBS.2010.5626430 21096143

[37] Mouysset S, Guivarch R, Noailles J, Ruiz D. Parallel spectral clustering for the segmentation of cDNA microarray images. 6^th^ International Conference on PACBB, AISC 2012;154:1–9.

[38] Giannakeas N, Karvelis PS, Exarchos TP, Kalatzi FG, Fotiadis DI. Segmentation of microarray images using pixel classification—Comparison with clustering-based methods. Computers in Biology and Medicine. 2013; 43(6):705–16. 10.1016/j.compbiomed.2013.03.003 23668346

[39] Giannakeas N, Karvelis PS, Fotiadis DI. A classification-based segmentation of cDNA microarray images using support vector machines. The 30th annual international IEEE EMBS conference. 2008; 875–8. 10.1109/IEMBS.2008.4649293 19162796

[40] Raghavarao S, Madhanmohan MS, Prasad GMV. Segmentation of microarray image using information bottleneck. Global Journal of Computer Science and Technology. 2011; 11(19):31–3.

[41] Larese MG, Granitto PM, Gómez JC. Spot defects detection in cDNA microarray images. Pattern Anal Applic. 2013; 16(3):307–19.

[42] Shao GF, Yang F, Zhang Q, Zhou QF, Luo LK. Using the maximum between-class variance for automatic gridding of cDNA Microarray Images. IEEE/ACM Transactions on Computational Biology and Bioinformatics. 2013; 10(1):181–92. 10.1109/TCBB.2012.130 23702554

[43] Rueda L, Rezaeian I. A fully automatic gridding method for cDNA microarray images. BMC Bioinformatics. 2011; 12:113–30. 10.1186/1471-2105-12-113 21510903 PMC3110145

